# The influence of professional identity on how the receiver receives and responds to a speaking up message: a cross-sectional study

**DOI:** 10.1186/s12912-023-01178-z

**Published:** 2023-01-30

**Authors:** Melanie Barlow, Bernadette Watson, Elizabeth Jones, Fiona Maccallum, Kate J. Morse

**Affiliations:** 1 Faculty of Health Sciences, Australian Catholic University, 1100 Nudgee Road, Banyo, QLD Australia; 2 School of Psychology, University of Queensland, St Lucia, QLD Australia; 3 Department of English and Communication, The Hong Kong Polytechnic University, Hung Hom, Kowloon, Hong Kong; 4 School of Psychology, Monash University Malaysia, Jalan Lagoon Selatan, 47500 Bandar Sunway, Selangor Darul Ehsan Malaysia; 5 College of Nursing & Health Professions, Drexel University, 245 N 15th Street, Mail Stop 501, 4th Floor, Room 4606, Philadelphia, PA 19102 USA

**Keywords:** Speaking up, Receiver, Communication accommodation theory, Healthcare communication, Safety voice, Social identity, Patient safety

## Abstract

**Background:**

Research focused on understanding what enables or hinders health professionals to speak up about a safety concern has been to date predominately atheoretical and speaker focused. However, the role the receiver of the message plays in these often-difficult encounters is highly influential. To date, speaking up programs have created conversational mnemonics that technically should respectfully engage the receiver, yet speaking up remains challenging. This paper utilises Communication Accommodation Theory to explore the impact the communication behaviour and speaker characteristics has on the receiver of a speaking up message, and if these impacts differ between receiver groups (clinical disciplines).

**Method:**

Clinicians (*N* = 208) from varying disciplines responded to two hypothetical speaking up vignettes, where participants were the receivers of speaking up messages. Analysis of variance was used to explore any potential differences between receiver groups.

**Results:**

Findings indicated that the level of perceived accommodation and group membership, whether defined by speaker discipline or seniority, collectively influenced how the receiver of a speaking up message evaluated the interaction, which influenced their anticipated response to the speaker.

**Conclusions:**

The receiver’s perceptions and evaluations of the message, their own professional identity and the presence of others, influenced receivers’ anticipated responses. This has direct implications on healthcare speaking up training and provision of care, as the varying clinical disciplines received and responded to the same messages differently.

**Supplementary Information:**

The online version contains supplementary material available at 10.1186/s12912-023-01178-z.

## Introduction

Failures in healthcare communication are well documented as a leading cause of medical error and patient harm [1]. A clinician’s ability and willingness to speak up to prevent or limit patient harm is a key contributing factor to enhancing both patient and staff wellbeing and safety [2]. Although there are a number of definitions of speaking up, key characteristics include voicing a concern up, down or across the hierarchical chain, with a focus on patient safety and staff physical and psychological safety [3, 4].

Despite knowing the importance of speaking up, many clinicians choose to remain silent and errors ensue [5]. As a result, speaking up communication encounters have been subject to a wide variety of studies which have identified many barriers and enablers [6, 7]. The anticipated response of the receiver of the message has shown to actively encourage or discourage speaking up, yet why they respond in the manner in which they do, has been largely unstudied. Our study, therefore, aimed to understand how receivers perceived a speaking up message in a defined clinical context, and what factors influenced their anticipated response.

## Background

Barriers and enablers to speaking up in healthcare are well defined, and include hierarchy and status differentials [8], leadership support and perceived psychological safety [5], organisational culture [9], and the predictability of the response, or fear of repercussions [6]. Organisations have developed and deployed speaking up programs to help staff initiate their voice and provide a structured framework to phrase their concerns. However, often these frameworks are generic, lack a structured and valid underlying communication theory, and rarely adapt the speaking up message to the clinical context, nor take account of differences in communication behaviour and expectations of the differing disciplines [10]. Moreover, very little research on speaking up has examined the receiver’s perspective, i.e., how the receiver of the speaking up message hears, processes, and responds in the moment during clinical care [11, 12]. Feedback literature supports the importance of how to receive feedback and manage defensiveness [13], and recommendations have been put forward for increased research into ‘hearer courage’ and ‘hearer action’ to enhance message reception within the context of whistleblowing [14]. Social psychology, through formal communication models, has always acknowledged the critical role of the receiver [15, 16]. Unfortunately, within healthcare, speaking up programs and associated research have not directly studied the receiver in this context, and therefore have fundamentally ‘best guessed’ what would be accepted by the receiver, which we argue is problematic. A more comprehensive understanding of how receivers perceive and respond to speaking up messages, underpinned by a sound theoretical model, is required to develop more targeted and effective communication training. We argue this focus will facilitate improved healthcare communication and ultimately patient outcomes.

Our study invokes Communication Accommodation Theory (CAT: [17]), a validated communication theory that has been used extensively within the healthcare context [18]. The theory is founded in social psychology and influenced by Social Identity Theory (SIT: [19]) and Attribution Theory [20]. CAT posits that each interactant comes to an interaction with their own motivations, and initial perceptions and feelings about the other person. These perceptions are influenced by factors such as the individual’s group memberships, interpersonal and intergroup history, and the social context [21]. Healthcare is steeped in traditional hierarchies [22] and has a strong intergroup environment (different disciplines, seniority levels, and specialties), each with their own cultural norms, directly impacting the efficacy of communication [23]. Thus, CAT is appropriate to use in our study.

Commencing in undergraduate training, health professionals are educated to, and develop professional identities e.g., nurse, doctor. As a result, each professional group develops unique attributes, world views and approaches to situations [24]. These social differences influence one group’s perception of the other, rather than their personal differences. The degree of acceptance of the other group’s social differences, is often in sociology referred to as the ‘social distance’ between the two [25]. CAT has been used to understand the intergroup dynamics between these differing health professionals, or ‘groups’, e.g., midwives and doctors [26], and within the same profession (medicine), but in differing seniority levels or subspecialties, e.g., gastroenterology and emergency medicine [27]. This intergroup behaviour is often discriminatory, competitive, and harmful [28], as seen in Setchell et al. [29] who, within endoscopy, explored communication encounters between medical officers and advanced practice nurses. The medical officers labelled the advanced practice nurses as incompetent, to reduce the threat to their medical officer professional identity. Speaking up in the literature is often framed as a challenging conversation. These intergroup dynamics, seniority and differing disciplines, are often cited as key challenges to voicing concerns [6, 30].

In addition to the group membership of the interactants, phrasing of the message and the perceived manner in which it is delivered, have been found to impact the receiver’s perceptions and evaluation of the speaker [31]. CAT describes how we accommodate or not (nonaccommodate) to a speech partner within an interaction, through what we say and how we say it. Each person in the interaction (speaker and receiver) can deploy specific communication strategies to reduce, maintain, or extend the social distance between them [32]. Where accommodation refers to strategies aimed to reduce the social distance to a speech partner, nonaccommodative strategies maintain or aim to increase the distance [33]. How a receiver hears, evaluates, and interprets a message can impact their ability and willingness to listen, and potentially widen the social distance, leading to miscommunication. This may directly impact patient safety, as well as the likelihood of engaging in, and the success of, future speaking up encounters for both the speaker and the receiver [5]. Our study focuses on how the accommodative or nonaccommodative stance of the speaker and their group membership (discipline and/or seniority) can influence a receiver’s perceptions of both the situation, and their communication behavioural intentions towards a speaker in a speaking up context.

In our previous study [34], we gained a foundational understanding of how the perceived level of accommodation by the speaker influenced real speaking up interactions, as described by the receiver of those messages. We found that speaking up encounters were very complex, as they were influenced by the group membership of profession and seniority, and speaker stance. The study demonstrated that current research on the person speaking up (speaker centric research) is not generalisable to the receiver within the same conversational context. More research is required to understand receiver evaluations and behaviour when the speaking up message is standardised across the receiver groups (all hearing the same message). Therefore, in the current study, we sought to understand the receivers’ perceptions, including their behavioural intentions, in a defined speaking up context, where the participants were receivers of a speaking up message. We used vignettes to focus on clinical speaking up encounters to understand what characteristics of the speaker (discipline, seniority, stance) influenced the receiver’s perceptions. The presence of an audience to the conversation (patient or colleagues) during a speaking up incident, has in previous speaking up studies been found to influence an individual’s communication behaviour [35]. We manipulated the speaker in the vignettes to investigate if this factor also impacted the receiver perceptions.

Despite believing there will be differences between receiver groups within this study, given the complexity of speaking up interactions and the paucity in receiver focused research, we believe it would be premature to put forward hypotheses as we are still unsure of how these three variables interplay. Therefore, we developed three overarching research questions.

- RQ1: When the speaking up interactions are defined within the vignettes, what characteristics of the speaker impact a receiver’s perceptions of a speaking up message?
- RQ2: How does the group membership (discipline) of the receiver influence their perceptions of a speaking up message?
- RQ3: How do the different receiver groups evaluate speaker stance?

## Methods

### Setting

We conducted our study at a metropolitan single site tertiary health service (800 beds), providing both private and public services. Data were collected between May 2019 and December 2019. The study had ethics approval from Mater Health Services Research Human Research and Ethics Committee HREC/18/MHS/78.

### Participants

Participants were recruited via a convenience sample at the commencement of a corporate training program which multiple disciplines attended. After 4 months of data collection, allied health and medical officers had lower than expected attendance and, therefore, a purposive sample was subsequently recruited during forums where there was protected time, e.g., workshops, in-service sessions, department meetings. A total of 208 clinicians (nurse/midwife: *n* = 142, allied health [physiotherapists, social workers, radiographers, pharmacists]: *n* = 44, medical officers [doctors]: *n* = 22) completed the survey over the eight-month data collection period. The overall response rate for survey completion across both samples was 44%. See Table 1 for participant characteristics.

**Table 1:** Participant characteristics

Characteristic	Nurse/Midwife		Allied Health		Medical Officer	
	*n*	%	*n*	%	*n*	%
	142	68.3	44	21.1	22	10.6
**Specialty**						
Critical Care	37	26.1	8	18.2	10	45.5
Perioperative	17	12.0	0	0	2	9.1
Inpatient Wards	63	44.4	19	43.2	6	27.3
Day Stay areas	2	1.4	1	2.3	0	0
Antenatal areas	2	1.4	3	6.8	0	0
Birth Suite	8	5.6	0	0	2	9.1
Outpatients	4	2.8	10	22.7	2	9.1
Interventional areas	5	3.5	3	6.8	0	0
Missing	4	2.8	0	0	0	0
**Years in Profession**						
3 years or less	65	45.8	13	29.5	10	45.5
4 to 8 years	32	22.5	10	22.7	2	9.1
9 to 14 years	14	9.9	10	22.7	7	31.8
15 to 20 years	11	7.7	4	9.1	1	4.5
More than 20 years	20	14.1	7	15.9	2	9.1
**Gender**						
Male	7	4.9	10	22.7	8	36.4
Female	135	95.1	34	77.3	14	63.6

### Materials and measures

We developed two vignettes portraying different hypothetical speaking up encounters for this study. As participants were from varying clinical disciplines, the hypothetical clinical situations had to be suitably generic and not discipline specific. Following consultation with different clinical cohorts, safety and quality personnel, and reviewing safety audits within the health organisation, two clinical situations were identified as common speaking up opportunities that all clinicians frequently encountered and struggled to effectively address. Vignette one involved nonadherence to Personal Protective Equipment (PPE) when caring for an infectious patient; the patient was present during the encounter. The second vignette involved nonadherence to the Hand Hygiene (HH) standard procedure, which occurred during a multidisciplinary patient round.

In the two vignettes, harm to the patient or staff member was not imminent. These ‘grey’ situations have been shown to be more difficult to speak up about [36], as there is less certainty about the impact, and therefore greater personal risk than a more definitive, black and white patient safety situation, e.g., wrong site surgery. In the vignettes, the clinician speaking up communicated their message in one of two ways, either using an accommodative or nonaccommodative manner. The accommodative message within the vignette used advocacy/inquiry [37] to frame the message. The nonaccommodative speaking up message was more abrupt and adopted a patronising tone. Moreover, it was framed as a statement rather than a question. See Table 2 for vignette examples.

**Table 2:** Receiver vignette examples

Nonaccommodative (PPE)	Accommodative (HH)
You are standing at the foot of the bed with your arms crossed in a room of a patient who is on contact precautions for MRSA. You have not put on the prescribed Personal Protective Equipment (PPE) as you only had to ask one question and you have ensured that you haven’t touched anything. One of the junior nurses/midwives puts their head through the door and states “*you need to get out now and put the appropriate PPE on, you know you should not be in here without it!*”	You are on the daily ward round in your unit as part of the interprofessional team and you have just moved from Mrs. Smith’s bed to Mrs. Williams, next door. You don’t believe you touched anything at Mrs. Smith’s bed space, however a senior medical officer on the ward round speaks up to you and in front of the interprofessional team, but not in front of the patient, and states “*ah, excuse me, I saw you coming to see Mrs. Williams after seeing Mrs. Smith and I didn’t see you wash or sanitise your hands. As part of the hand hygiene procedure, this needs to be done before you see Mrs. Williams in order to protect our patients. I’m just wondering what’s up?*”

**Fig. 1:**
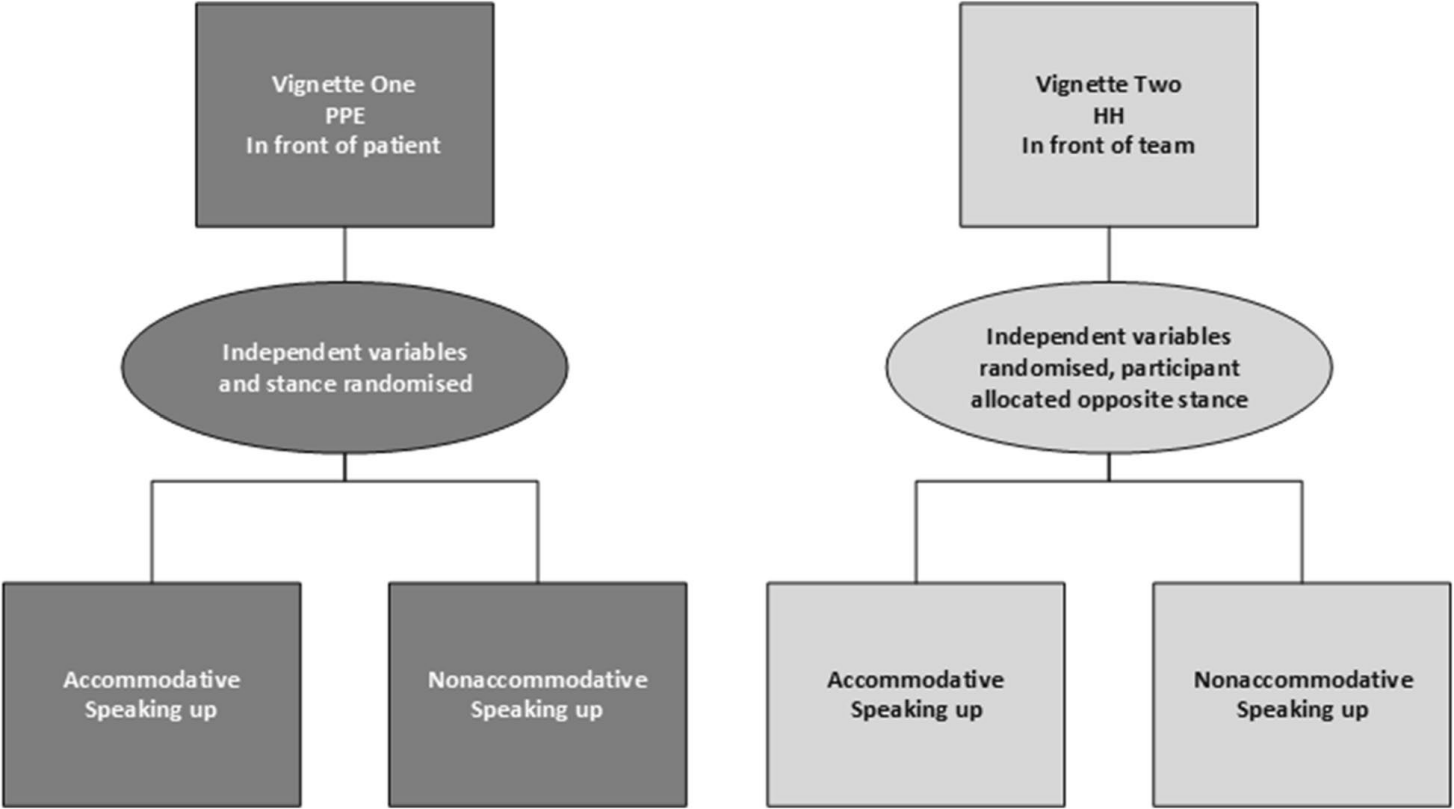
Organisation of vignette variables

In the PPE vignette, speaking up occurred in the presence of the patient and in the HH vignette, in the presence of the team. The characteristics of the person speaking up in the vignette that were varied were seniority (senior, junior), discipline (nursing/midwifery, allied health, medical officers), and communication behaviour [stance] (accommodative, nonaccommodative). Each participant was presented with both vignettes with all variables (seniority, discipline and stance) randomised and counterbalanced across the two vignettes, see Fig. 1. Thereby, each participant completed one accommodative and one nonaccommodative vignette. Randomisation of the variables across the two clinical situations (PPE, HH), resulted in 24 unique vignettes.

Participants rated both vignettes using the same set questions, which focused on their perceptions and evaluations of the speaker. Questions included the receivers’ perceptions about the acceptability of the message, their level of discomfort, the effect of discipline of the speaker and the context of the vignette with a patient or team present. Participants responded to each question on a Likert-type scale (1 = Strongly disagree to 7 = Strongly agree). These questions were developed from a review of the speaking up literature for frequently reported barriers and enablers to speaking up. The questions sought to ascertain the receiver’s response to being spoken up to in relation to these barriers (seniority, differing disciplines, existing relationships, how the message was delivered, and the presence of others).

The clinical scenarios and survey questions were reviewed by a panel of experts; international experts in CAT, experienced clinicians from different disciplines, and experts in the use of advocacy/inquiry based questioning, for readability, and real world and theoretical relevance.

### Procedure

Participants who consented were provided with the paper-based survey. Each participant completed two counter-balanced vignettes. Survey results were entered into an Excel spreadsheet. Ten percent of the data was double entered to check for accuracy, which was deemed to be accurate with an error rate of 0.7% [38].

### Data analysis

We analysed the data using SPSS statistical software. Multifactorial Analysis of Variance (ANOVA) where the independent variables were between speaker discipline groups (nursing/midwifery, allied health, or medical officer), stance (accommodative or nonaccommodative), and speaker seniority levels (junior or senior to the participant), resulting in a 3x2x2 between-subjects ANOVA. The dependent variables were the scores the from each of survey questions. Ten of the eleven survey questions were analysed. The eleventh question was a short answer question, results of which are reported elsewhere [39]. Analyses were conducted separately for each of the three receiver groups (nursing/midwifery, allied health, medical officer), allowing for comparison of the results for different disciplines. This was done to ascertain if, or how, the receiver’s discipline (group membership) influenced their perceptions. Owing to the differences in sample size for participant disciplines, robust analysis methods with bootstrapping were applied. Bootstrapping reduces possible bias when assumptions, such as equal group sizes, have been violated [40, 41]. The bootstrapping for each independent variable took one thousand (1000) samples from the data set and used a 95% confidence interval via a bias corrected accelerated confidence interval (BCa). A *p* value of less than 0.05 was considered statistically significant. Bonferroni correction was applied for multiple comparisons. Effect size was measured using Partial Eta squared (*η*^2^).

For the statistical analysis of the survey items, an a-priori power analysis was conducted using G*Power [42] with power 0.8, a medium partial effect size (*η*^2^) of 0.06 (effect size f 0.25) and alpha 0.05. The minimum sample size to determine a significant difference between the groups, if one exists, was estimated to be 155 participants. To understand the influence of receiver discipline, data was analysed by receiver group (nursing/midwifery, allied health, medical officers), therefore a minimum of 50 in each group was required. Initial analyses found that the two vignettes were rated similarly for both the accommodative and nonaccommodative versions, therefore to ensure a sufficient sample size, the participants’ ratings of the two vignettes were analysed together.

## Results

The target sample size was 155 and a total sample of 208 was achieved. As each participant completed two vignettes, a total of 416 observations for analysis was obtained. However, the desired power for medical officers was not reached.

**Fig. 2:**
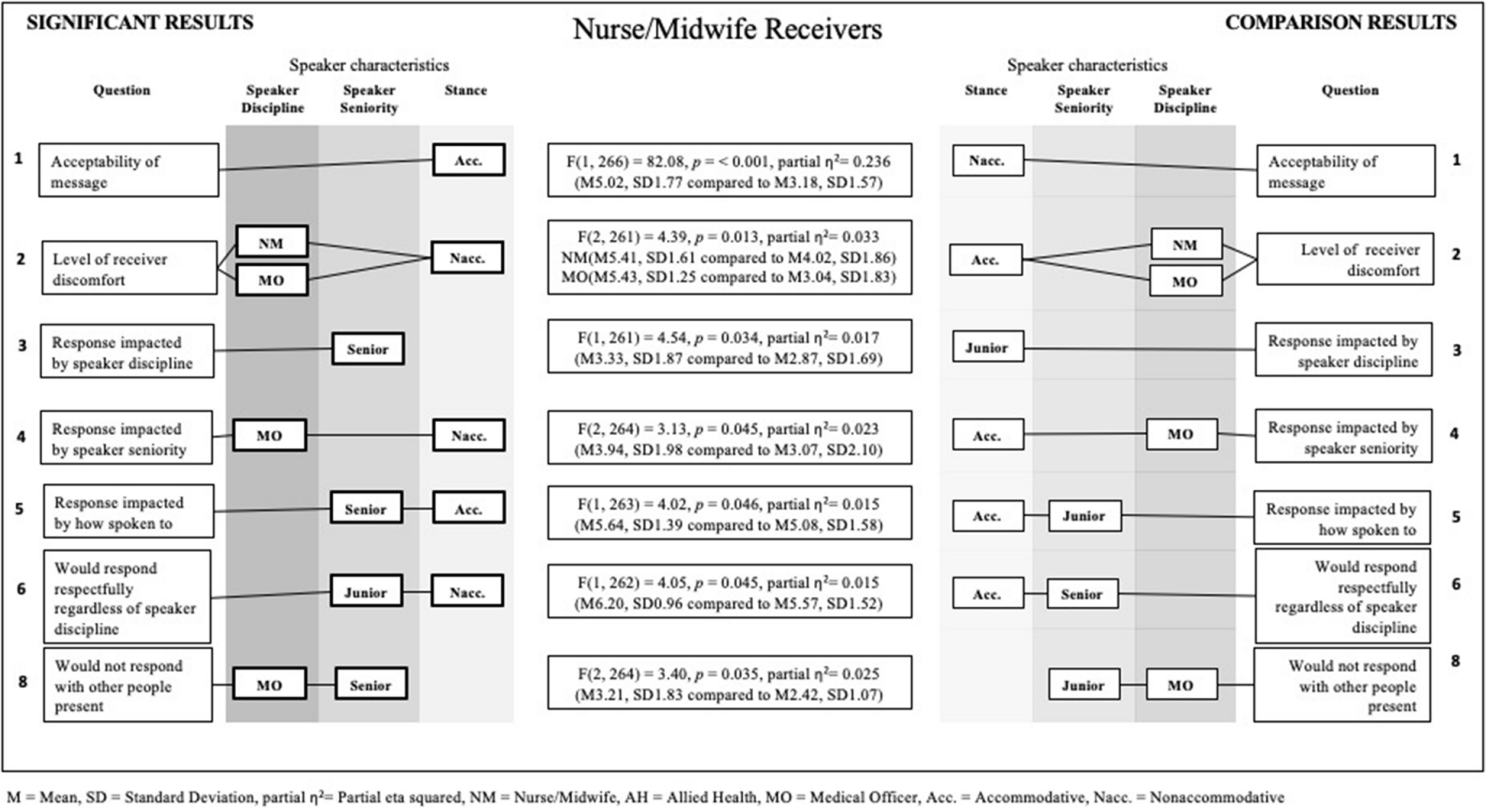
Significant results for nurse/midwife receivers

**Fig. 3:**
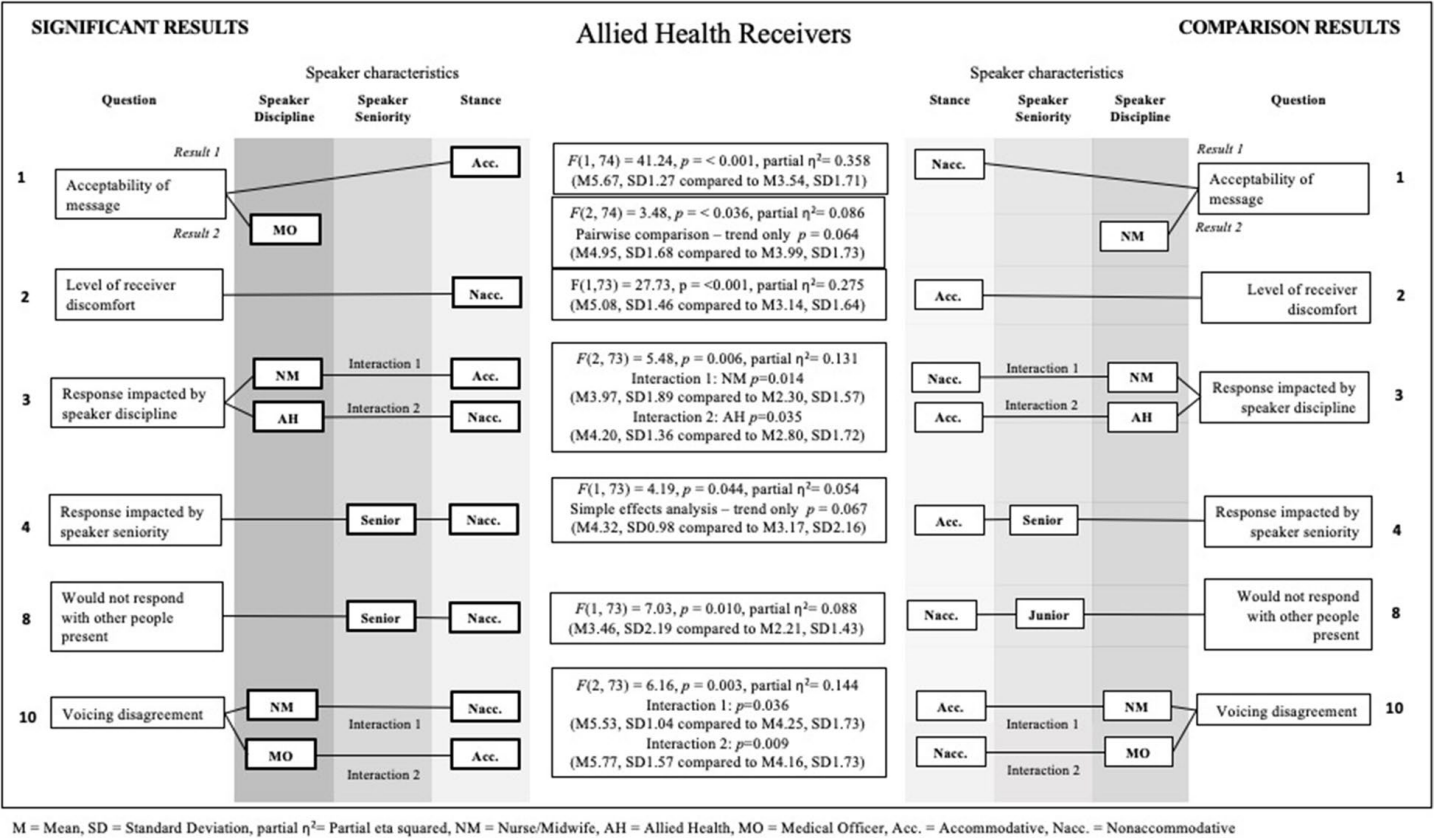
Significant results for allied health receivers

**Fig. 4:**
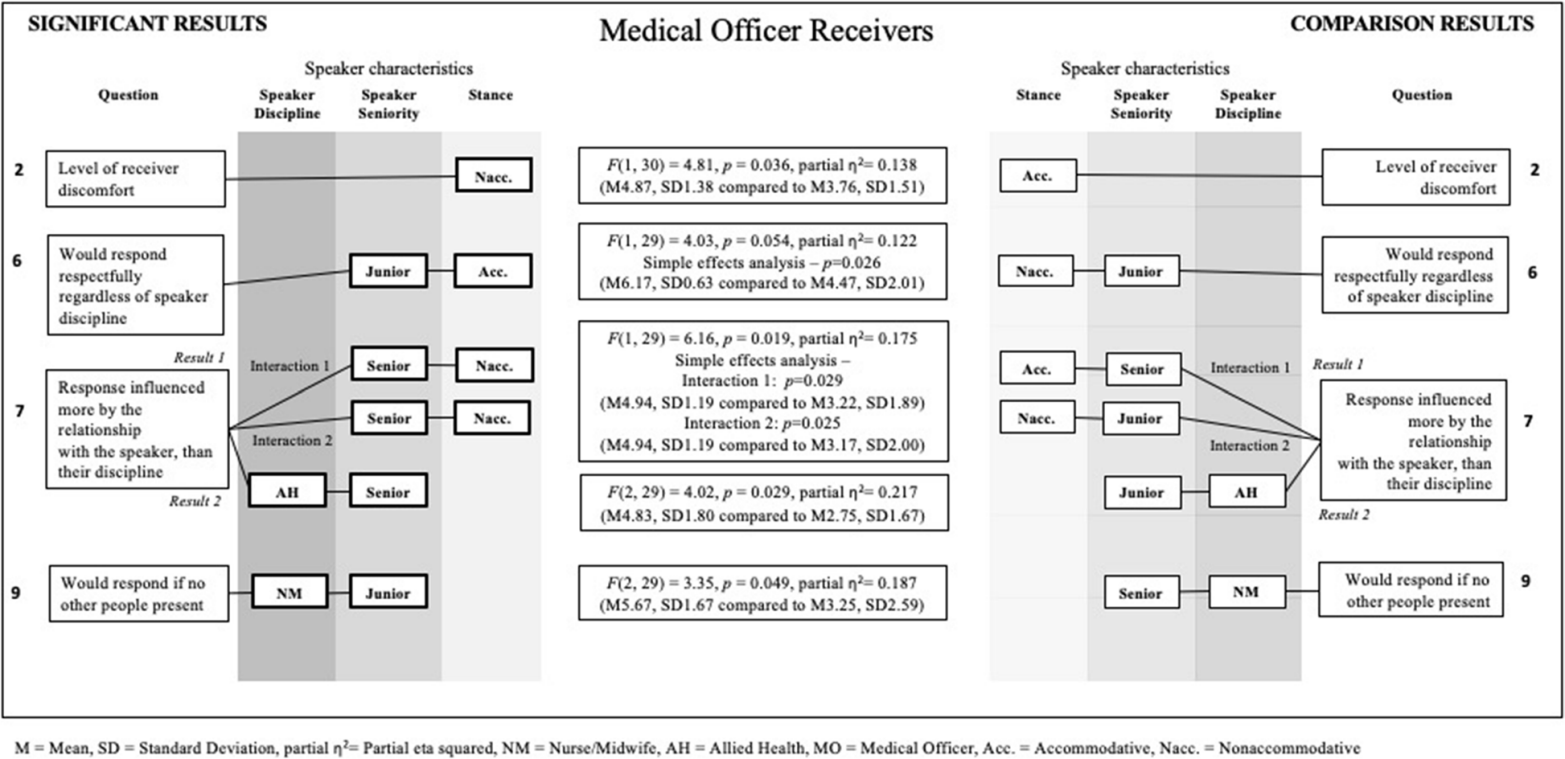
Significant results for medical officer receivers

A 3 × 2 × 2 between-subjects (speaker discipline, speaker seniority and stance) ANOVA was conducted for each of the three receiver discipline groups, for each dependent variable (question items). Where an interaction effect occurred, simple effects analysis was undertaken for all interactions to see where the significant interaction was occurring. To aid interpretation, the significant findings are presented in Fig. 2 for nurses/midwives, Fig. 3 for allied health and Fig. 4 for medical officers. See supplementary file for a summary of means and standard deviations for each independent variable across the survey items.

### Q1: In this situation, the communication behaviour of the person who spoke up to me was acceptable, considering the circumstances

There was a significant main effect for stance for both the nursing/midwifery *F*(1, 266) = 82.08, *p* = < 0.001, partial *η*^2^ = 0.236. and allied health *F*(1, 74) = 41.24, *p* = < 0.001, partial *η*^2^ = 0.358 receiver groups. Nurse/midwife and allied health receivers rated the message as significantly more acceptable when the speaker used an accommodative stance (nurse/midwife: M = 5.02, SD = 1.77; allied health M = 5.67, SD = 1.27), compared to when the speaker was nonaccommodative (nurse/midwife: M = 3.18, SD = 1.57; allied health M = 3.54, SD = 1.70). However, medical officers did not rate one speaker stance as being significantly more acceptable than the other (*p* = 0.236, nonaccommodative M = 3.94, SD = 1.77; accommodative M = 4.72, SD = 1.65). This nonsignificant difference in stance for medical officers indicates they found both stances reasonably acceptable.

### Q2: In this situation, the communication behaviour of the person who spoke up to me made me feel uncomfortable

There was a significant main effect for stance for nursing/midwifery receivers (*p* = < 0.001), which was subsumed by a two-way interaction between stance and speaker discipline *F*(2, 261) = 4.39, *p* = 0.013, partial *η*^2^ = 0.033. Nurse/midwifery receivers rated significantly higher discomfort levels when receiving a nonaccommodative message from a medical officer (*p* = < 0.001, M = 5.43, SD = 1.25) and a fellow nurse/midwife (*p* = < 0.001, M = 5.41, SD = 1.61), compared to an accommodative medical officer (M = 3.04, SD 1.83) and nurse/midwife (M = 4.02, SD = 1.86). There was no significant difference for nurse/midwife receivers when the speaker was allied health (accommodative *p* = < 0.939, M = 4.05, SD = 1.97; nonaccommodative *p* = < 0.188, M = 4.95, SD = 1.52).

A significant main effect for stance was found for allied health *F*(1, 73) = 27.73, *p* = < 0.001, partial *η*^2^ = 0.275, and medical officers *F* (1, 30) = 4.81, *p* = 0.036, partial *η*^2^ = 0.138, (M = 4.87. Receivers rated significantly higher discomfort levels when the stance was nonaccommodative (allied health: M = 5.08, SD = 1.46; medical officers M = 4.87, SD = 1.38) compared to accommodative (allied health: M = 3.14, SD = 1.64; medical officers M = 3.76, SD = 1.51).

### Q3: In this situation, how I respond to the person who spoke up to me would be influenced by what discipline they are

The ANOVA for nurse/midwifery receivers identified a significant main effect of speaker seniority *F*(1, 261) = 4.54, *p* = 0.034, partial *η*^2^ = 0.017. Receivers gave a significantly higher influence rating for the speaker discipline when the speaker was more senior (M = 3.33, SD = 1.87) compared to when the speaker was more junior (M = 2.87, SD = 1.69).

The ANOVA for allied health receivers identified a significant two-way interaction between speaker discipline and stance *F*(2, 73) = 5.48, *p* = 0.006, partial *η*^2^ = 0.131. Analysis revealed two effects. Allied health receivers gave a significantly higher influence rating when the speaker was an accommodative nurse/midwife (M = 3.97, SD = 1.89), compared to a nonaccommodative nurse/midwife (M = 2.30, SD = 1.57). Additionally, allied health receivers gave a significantly higher influence rating when the speaker was a nonaccommodative allied health colleague (M = 4.20, SD = 1.36), compared to an accommodative allied health colleague (M = 2.80, SD = 1.72).

For medical officer receivers, there were no significant differences in influence ratings for speaker discipline. Means indicate speaker discipline was not an influencing factor for medical officers responding to either an accommodative, or nonaccommodative speaking up message (Speaker discipline: nurse/midwife M = 3.24, SD = 1.60, allied health M = 2.50, SD = 1.34, and medical officer M = 2.98, SD = 1.63).

### Q4: In this situation, how I respond to the person who spoke up to me would be influenced by how senior they are

The ANOVA for nurse/midwife receivers identified a significant two-way interaction between speaker discipline and stance *F*(2, 264) = 3.13, *p* = 0.045, partial *η*^2^ = 0.023. Nurse/midwifery receivers gave significantly higher influence ratings for speaker seniority when the speaker was a nonaccommodative medical officer (M = 3.94, SD = 1.98) compared to an accommodative medical officer (M = 3.07, SD = 2.10). There was no significant difference for the influence of seniority when the speaker was from a nursing/midwifery or allied health discipline.

The ANOVA for allied health receivers identified a significant two-way interaction between speaker seniority and stance *F*(1, 73) = 4.19, *p* = 0.044, partial *η*^2^ = 0.054. Simple effects analysis indicated a trend only (*p* = 0.067). Allied health participants tended to give higher influence ratings for speaker seniority when the speaker was a senior speaking nonaccommodatively (M = 4.32, SD = 0.98) compared to a senior speaking accommodatively (M = 3.17, SD = 2.16). There was no significant difference in response ratings by allied health professionals for junior speakers.

For medical officer receivers, there were no significant differences in influence ratings for speaker seniority. Means indicate that for medical officers, speaker seniority (junior M = 3.12, SD = 1.84; senior M = 3.46, SD = 1.63) was not a strong influence with respect to their response to either an accommodative, or nonaccommodative speaking up message.

### Q5: In this situation, how I respond to the person who spoke up to me would be influenced by how they spoke to me (tone of voice, the words they use)

The ANOVA for nurse/midwife receivers identified a significant two-way interaction between speaker seniority and stance *F*(1, 263) = 4.02, *p* = 0.046, partial *η*^2^ = 0.015. The receivers gave significantly higher influence ratings about how they anticipated they would respond when the message was delivered when the speaker was an accommodative senior (M = 5.64, SD = 1.39), compared to an accommodative junior (M = 5.08, SD = 1.58). However, there was no difference for a nonaccommodative speaker.

There was no significant difference in the influence ratings between either allied health and medical officer receivers.

### Q6: In this situation, I would respond respectfully regardless what discipline the person is, who is speaking up to me

The ANOVA identified a significant significant main effect for nursing/midwifery receivers for seniority (*p* = 0.021), which was subsumed by a two-way interaction between speaker seniority and stance *F*(1, 262) = 4.05, *p* = 0.045, partial *η*^2^ = 0.015. Nurse/midwife receivers rated that they were significantly more likely to respond respectfully regardless of the speaker’s discipline, if the speaker was an accommodative junior (M = 6.20, SD = 0.96), compared to an accommodative senior (M = 5.57, SD = 1.52). There was no significant difference for respect for speaker discipline when the stance was nonaccommodative. There was no significant difference in ratings for respectful responding across stance or seniority for allied health receivers.

The ANOVA for medical officer receivers identified a trend for a two-way interaction effect for speaker seniority and stance *F* (1, 29) = 4.03, *p* = 0.054, partial *η*^2^ = 0.122. This trend had a large effect size, therefore a simple effects analysis was undertaken. Medical officer receivers rated that they were significantly more likely to respond respectfully regardless of the speaker’s discipline (*p* = 0.026) if the speaker was an accommodative junior (M = 6.17, SD = 0.63), compared to a nonaccommodative junior (M = 4.47, SD = 2.01). This suggests that they would be less respectful to a nonaccommodative junior. There was no significant difference in their rating of respectful response for stance when the speaker was senior (accommodative senior M = 5.75, SD = 1.75; nonaccommodative senior M = 6.00, SD = 0.98), indicating they would always be respectful.

### Q7: Regardless of the situation, how I respond to the message is influenced by my relationship with that person, not what discipline they are

There were no significant differences across stance in the influence ratings for their relationship with the speaker for both nurse/midwife (accommodative M = 3.89, SD = 1.30; nonaccommodative M = 3.78, SD = 1.10), and allied health receivers (accommodative M = 3.58, SD = 0.99; nonaccommodative M = 3.99, SD = 1.23).

The ANOVA for medical officer receivers identified two significant two-way interactions, for speaker seniority and stance *F* (1, 29) = 6.16, *p* = 0.019, partial *η*^2^ = 0.175 and speaker discipline and seniority *F* (2, 29) = 4.02, *p* = 0.029, partial *η*^2^ = 0.217. Analyses of simple effects for speaker seniority and stance found that medical officer receivers rated that they were significantly more likely to be influenced by the relationship with the speaker when the speaker was a nonaccommodative senior (M = 4.94, SD = 1.19), compared to both an accommodative senior (M = 3.22, SD = 1.89) and a nonaccommodative junior (M = 3.17, SD = 2.00). For the interaction between speaker discipline and seniority, medical officer receivers gave significantly higher influence rating for relationship with the speaker when the speaker was a senior allied health member (M = 4.83, SD = 1.80), compared to a junior allied health member (M = 2.75, SD = 1.67). There were no significant differences in the influence ratings when the speaker was a nurse/midwife (junior M = 3.60, SD = 1.91; senior M = 4.50, SD = 1.80), or medical officer (junior M = 4.75, SD = 1.67; senior M = 2.92, SD = 1.58). Overall medical officer responses are impacted by the relationships when the speaker is a nonaccommodative senior, or a senior allied health member.

### Q8: In this situation, with other people being present in the room, I would not respond to the person who spoke up to me

The ANOVA for nurse/midwife receivers identified a significant two-way interaction between speaker discipline and speaker seniority level *F*(2, 264) = 3.40, *p* = 0.035, partial *η*^2^ = 0.025. Nurses/midwives rated that they were significantly more likely to remain silent when spoken to in the presence of others when the speaker was a senior medical officer (M = 3.21, SD = 1.83), compared to when the speaker was a junior medical officer (M = 2.42, SD = 1.07). There was no significant difference when the speaker was allied health or another nurse/midwife. This suggests that senior medical officers inhibit nurse/midwife intention to speak when others are present.

The ANOVA for allied health receivers identified a significant two-way interaction between speaker seniority level and stance *F*(1, 73) = 7.03, *p* = 0.010, partial *η*^2^ = 0.088. Allied health receivers rated that they were significantly more likely to remain silent when spoken to in the presence of others when the speaker was a nonaccommodative senior (M = 3.46, SD = 2.19), compared to nonaccommodative junior (M = 2.21, SD = 1.43). There was no significant difference when the stance was accommodative. Allied health reported being inhibited by a senior speaker’s nonaccommodative stance.

There were no significant differences in the ratings of being silent for medical officer receivers in the presence of others regardless of stance (accommodative M = 1.95, SD = 1.07; nonaccommodative M = 2.10, SD = 1.41). The mean scores indicate they rated they would not remain silent regardless of stance, or indeed discipline or seniority.

### Q9: In this situation, I would respond to the person who spoke up to me if no other people were in the room

There were no significant differences in how receivers would respond if no other people were in the room for both nurse/midwife (accommodative M = 4.47, SD = 1.92; nonaccommodative M = 4.76, SD = 1.83), and allied health (accommodative M = 5.39, SD = 1.83; nonaccommodative M = 5.02, SD = 1.87) receivers, regardless of stance.

The ANOVA for medical officer receivers identified a significant two-way interaction between speaker discipline and speaker seniority level *F* (2, 29) = 3.35, *p* = 0.049, partial *η*^2^ = 0.187. Medical officers rated that they were more likely to respond to a junior nurse/midwife (M = 5.67, SD = 1.67) if other people were not present, compared to a senior nurse/midwife (M = 3.25, SD = 2.59). There was no significant difference in their agreement of whether they would respond when the speaker was allied health (junior M = 4.29, SD = 2.40; senior M = 4.92, SD = 1.86), or another medical officer (junior M = 3.37, SD = 2.59; senior M = 5.25, SD = 1.81).

### Q10: in this situation, if I didn’t agree with the person speaking up, I would say so

The ANOVA identified no significant differences regardless of stance, on their rating of speaking up for both nurse/midwife (accommodative M = 4.68, SD = 1.55; nonaccommodative M = 5.00, SD = 1.40) and medical officer receivers (accommodative M = 4.44, SD = 2.02; nonaccommodative M = 4.47, SD = 2.14). Means indicate that these receivers rated they would likely voice their disagreement with the speaker.

The ANOVA for allied health receivers identified a significant two-way interaction between speaker discipline and stance *F*(2, 73) = 6.16, *p* = 0.003, partial *η*^2^ = 0.144. Analyses of simple effects identified that allied health receivers rated that they were significantly more likely to voice disagreement with a nonaccommodative nurse/midwife (M = 5.53, SD = 1.04), compared to an accommodative nurse/midwife (M = 4.25, SD = 1.73). Additionally, allied health participants rated that they were significantly more likely to voice disagreement with an accommodative medical officer (M = 5.77, SD = 1.57), compared to a nonaccommodative medical officer (M = 4.16, SD = 1.73). There was no significant difference in their speaking up rating when the speaker was allied health (accommodative M = 5.37, SD = 1.11; nonaccommodative 5.33, SD = 1.61).

## Discussion

Our study aimed to understand the influence of speaker characteristics, receiver group membership (discipline and seniority) and stance on a speaking up message, have on the receiver’s anticipated behaviour in speaking up interactions defined within vignettes. As noted, we did not propose hypotheses. Our findings confirm our belief that these interactions are very complex and strongly intergroup in nature. The speaker’s role and behaviour (i.e., stance, seniority and/or discipline), and the receiver’s professional identity, influenced message reception and receivers’ intended response. It was also apparent that presence of others impacts on the receiver’s response.

In answering the research questions, the differing receiver groups evaluated and were influenced by speaker characteristics (stance, discipline and seniority) differently. For nurse/midwifery receivers, we found that stance, seniority, and the speaker being a medical officer, influenced their perceptions about how they received the message, and their anticipated response. Across the results, the speaking up message was rated as more acceptable, and nurse/midwife receivers rated that they experienced less discomfort when the stance was accommodative, compared to nonaccommodative. Receiving messages from a nonaccommodative medical officer or fellow nurse/midwife, caused significant receiver discomfort within this group. Nurse/midwife receiver voice was also shown to be significantly inhibited by senior medical officer speakers when other people were present during the interaction.

The seniority of the speaker, whether by years of experience or profession, was also a key factor influencing nurse/midwife perceptions about their intended response, particularly when it interacted with the use of a nonaccommodative stance. Speaker centric research has identified that nurses/midwives struggle to voice their concerns up the hierarchy, whether that hierarchy is based on level of experience (years), or as defined by traditional discipline hierarchical structures (medical officers in the position of dominance and power) [8, 9, 43]. Our results found that nurse/midwife receivers reported that they were not significantly affected by a junior person speaking up, as speaker-centric research discusses and anticipates. For receivers, it is the messages down the hierarchical chain, that is, when the message is delivered by a more senior person (seniority by years or discipline), that had a significant effect on the anticipated receiver responses. These results support the findings in a recent qualitative analysis of receiver behaviour [34], where receivers of speaking up messages, regardless of speaker stance, had more negative emotional reactions to senior speakers, than those who were more junior. This demonstrates that speaker centric research findings do not mirror the experiences and perceptions of receivers of the message. The results unfortunately highlight that within speaking up conversations, whether speaking or receiving, being a junior nurse/ midwife is a difficult position to be in.

Like nurse/midwife receivers, allied health rated that they preferred the accommodative stance to the nonaccommodative, finding it more acceptable and comfortable to receive. The key difference between allied health and nurse/midwife receivers was how their anticipated response rating was also influenced by speaker discipline. Allied health receivers indicated that their intended response was influenced more by accommodative intergroup and nonaccommodative ingroup interactions, compared to nonaccommodative intergroup and accommodative ingroup interactions. This demonstrates that it is not only how, but also who delivered the message that influenced their ratings of their intended response. Allied health is comprised of a collective of different health professionals of varying scopes of practice, delivery models and philosophical approaches [44], where interprofessional communication may include communication with other disciplines within allied health as well as medical officers and nurses/midwives. Additionally, allied health professionals often work in isolation, rather than in a team, e.g., one physiotherapist may be assigned to work a shift within an intensive care unit. Thus, a physiotherapist may work with the bedside nurse rather than with another physiotherapist, or allied health member in the provision of care. Therefore, given that clinical communication frequently occurs between different discipline groups, it is unsurprising to see more effects for discipline for allied health receivers.

When investigating the effect of receiver discipline, allied health receivers were the only discipline that reported they would respond respectfully regardless of how they were spoken to. We found that allied health receivers perceived they were more likely to respond respectfully when the speaker’s stance was accommodative than nonaccommodative, aligning with how allied health evaluated the acceptability of the message and level of comfort. We don’t yet know how both the speaker and receiver define what a respectful response is. However, this finding implies that if the receiver of the speaking up message feels the speaker is making an effort (being accommodating), they are more inclined to respond in an accommodative manner [32, 45]. A recent qualitative analysis of speaking up message reception, [39] identified that allied health receivers made the most positive attributions about the speaker, positively influencing message reception. Allied health receivers were able to do this more than any other discipline, especially when speaker stance was nonaccommodative. One possible reason may be due to allied health having to constantly negotiate with, and accommodate to, other health disciplines in order to achieve their planned care e.g., negotiating with bedside nurse for time with the patient, and seeking medical referrals. With comparative paucity of allied health speaking up literature, further research is required to help understand drivers of allied health behaviour in the speaking up context.

Overall, all receiver groups perceived reduced discomfort when receiving the accommodative message than the nonaccommodative. However, what was not expected was that unlike nurses/midwives and allied health, medical officer ratings did not find the accommodative messages significantly more acceptable than the nonaccommodative. This unexpected finding suggests that medical officers may evaluate how a message should be delivered differently than the other disciplines, and are less concerned with accommodative stance. It is not that medical officers did not feel discomfort, they clearly did from the results, but rather they may view their discomfort as less important than the message itself, and its level of perceived acceptability. This finding has consequences for intergroup speaking up encounters. A difference in opinion of what is an appropriate speaking up message has implications not only for how receivers hear the message and respond, but also how clinicians are trained to speak up. It is possible that medical officers prefer shorter, sharper messages that have traditionally been viewed as nonaccommodative. When looking through the lens of another theoretical perspective, medical officers have greater social and cultural capital (symbolic capital) within the healthcare system, than other professional groups [46, 47]. Being in a position that has traditionally been perceived as having legitimate positional power, according to social capital theory, gives the group symbolic domination [48]. That is, by virtue of being a doctor (social capital) puts them in a higher class category (cultural capital) [46, 49]. This provides the group greater access to organisational resources, trust and recognition [48]. Being in such a position may explain why medical officer behaviour in our study was not impacted by the presence of others, or how the message was delivered, as they hold greater social and cultural capital. This was identified in a recent publication studying the speakers perceptive, where positions with greater symbolic capital e.g., senior health professionals, found speaking up easier than those with lower symbolic capital e.g., junior health professionals [46]. Further research is required with a larger sample size for medical officers to explore this difference and its potential implications for interprofessional communication.

Interestingly, medical officers were the only receiver group whose ratings indicated that their relationship with the speaker would influence their response rather than speaker discipline. This relationship was significant when receiving a nonaccommodative message from a senior, of any discipline, compared to a junior. Means indicated that regardless of stance, the relationship with a senior medical officer in particular had the least influence on medical officer response. This finding was unusual, as literature has identified the difficulty junior doctors face when having to speak up to a senior of the same discipline [30]. Peadon’s systematic review found relationships with a more senior medical officer was as an important factor for career progression. Supported by Belyansky et al. [50], medical trainees felt more comfortable speaking up to a senior with whom they had a relationship. When looking further at our study results, it was seen that medical officer receivers identified that they would consistently be respectful when responding to a senior. This indicates that regardless of their relationship status with their senior, medical officer receiver response would be consistently respectful/accommodating.

Unlike nurses/midwives, the presence of others did not inhibit medical officer receiver response. Results indicated that this receiver group would voice their disagreement and would not remain silent when others were present in the room. This is consistent with the findings in a related study [39] where the presence of others was not identified as a concern for doctors but was a substantial concern for nurses/midwives.

Our study highlights how the receivers’ perceptions of a speaking up message are influenced by the speaker’s hierarchical status (discipline and/or seniority) and their perceived level of accommodation. This finding is consistent with other research regarding intergroup dynamics within the healthcare context [26]. It can be seen from the results that speaking up communication is complex. Speaker stance, seniority and discipline influenced the perceptions of the receiver groups differently, as did the presence of others. Our results suggest that speaking up training should be interprofessional in order to understand and appreciate differing communication behaviours that are specific to particular disciplines, and understand the impact that their behaviour has on the perceptions and behavioural intentions of other discipline groups. Furthermore, the presence or absence of others needs more investigation. This paper has provided further information on receiver behaviour supporting future studies to start predicting receiver response in certain contexts.

### Implications for practice and future research

When looking at the impact of the speaker’s seniority across all receiver groups, the results indicate that receivers agreed that the senior speaker more frequently influenced the receiver’s perceptions and anticipated response than a more junior speaker. The possibility that senior receivers are not as opposed to a junior speaking up as previously anticipated or presumed by speakers [8, 36, 51], could significantly lower a commonly identified barrier for junior clinicians voicing a concern. Organisational culture considerations need to be taken into account, as this organisation did have a hospital wide speaking up program in place, which could have positively influenced communication behaviour.

The organisation’s training program, like most others, teaches that regardless of who you need to speak up to (seniority or discipline), a prescribed speaking up mnemonic should be initiated when a concern needs to be raised. There are many advantages to having a shared language through a standardised mnemonic, but a universal mnemonic does not address, or even acknowledge the intergroup dynamics in these challenging conversations. Currently, most reported studies regarding speaking up training are focused on a single discipline [52]. Our study suggests that speaking up training programs need to be interprofessional in order for both speakers and receivers to understand and appreciate the impact of the intergroup dynamics within these conversations, and how to manage them within the speaking up interaction. In this way, there may be more focus and emphasis on engaging in a conversation, rather than the skill of remembering and implementing a mnemonic.

The healthcare sector needs to better understand the receiver’s perceptions and behaviour through the lens of communication theories, informed observational studies, and greater understanding of the receiver’s lived experience. This is essential to start tangibly and meaningfully applying communication strategies within training programs, to equally support and train both the speaker and the receiver.

### Limitations

The clinical and speaking up conversational contexts in this study were based on real situations within the organisation and are situations faced by many clinicians regularly in healthcare, making the speaking up concerns generalisable to the tertiary healthcare setting; this is the great strength of the paper. Nonetheless it is a limited set of speaking up encounters. This study had difficulty engaging medical officers to complete the survey, resulting in a lower than desired sample size, and therefore the results should be viewed with caution. However, the medical officer significant results had medium to large effect sizes (*η*^2^. 0122 − 0.217) indicating potentially strong practical implications of the findings, and therefore, warrant consideration and further investigation in future studies. Data collection could not be extended due to conflicting scheduled research activities in the organisation, and with the onset of COVID-19 in early 2020.

This study did have an over representation of female participants which is potentially due to the sampling strategy. Healthcare does however have a greater proportion of females to males, due to the composition of the largest health professional workforce; nursing and midwifery. Studies have shown that gender does impact voice within nursing, due to its predominant female workforce and associated gender role expectations [53]. Interestingly, a study using hypothetical scenarios via vignettes by Schwappach and Gehring [54], found a reduced likelihood of speaking up specifically within junior male nurses. However, a recent large, multisite study across health disciplines found that overall, males reported higher levels of confidence in, and greater organisational support for speaking up than females [46]. We do acknowledge that receiver gender has the potential to influence message reception and response. This study did not take receiver gender into consideration for two reasons. Firstly, this study was focused on the impact of speaker characteristics (manipulated within the vignettes), and receiver discipline. Secondly, due to the comparably low number of male participants, the authors felt that meaningful comparisons between receiver genders could not be made. We do however strongly encourage future receiver focused studies to consider the influence of gender.

## Conclusion

Speaking up research to date has overwhelmingly focused on the person speaking up. Our findings clearly demonstrate that the receiver of a message is also influenced by the person they are interacting with, the speaker’s communication behaviour, their own professional identity and the presence of others. This suggests that speaking up training needs to stop being predominately based on technical skill (learning a mnemonic) and theorising how receivers want to be spoken to. To help advance our knowledge of receiver behaviour, future studies are in train to better understand situational context, and further explore intergroup dynamics in both self-reported data and observed receiver behaviour.

## Supplementary Information

**Additional file 1.** Summary of overall means and standard deviations for independent variables.

## Data Availability

All data generated or analysed during this study are included in this published article and its supplementary files expect for one question. The question was removed from analysis as it did not add any additional information to the findings and was written in a way that could result in a strong social desirability bias. This additional data is available from the corresponding author upon reasonable request.
