# Supplementary material for: The influence of professional identity on how the receiver receives and responds to a speaking up message: a cross-sectional study
Source: BMC Nurs. 2023 Jan 30;22:26. doi: 10.1186/s12912-023-01178-z (PMC9884599; doi:10.1186/s12912-023-01178-z)
Supplement: Supplementary file 1 — Additional file 1. Summary of overall means and standard deviations for independent variables. [file 12912_2023_1178_MOESM1_ESM.docx]

Supplementary File

*Summary of overall means and standard deviations for independent variables*

|  | | Study participants (Receivers) | | | | | |
| --- | --- | --- | --- | --- | --- | --- | --- |
|  | | Nurse/midwife | | Allied health | | Medical officers | |
| Survey question | **Speaker characteristics** | **Mean** | **SD** | **Mean** | **SD** | **Mean** | **SD** |
| 1 | Stance – accommodative  – nonaccommodative | 5.02  3.18 | 1.77  1.57 | 5.67  3.54 | 1.27  1.70 | 4.72  3.94 | 1.65  1.77 |
|  | Discipline – nurse/midwife  – allied health  – medical officers | 3.86  4.18  4.27 | 1.94  1.85  1.92 | 3.99  4.89  4.95 | 1.68  1.98  1.73 | 4.82  4.27  3.90 | 1.50  1.75  2.02 |
|  | Seniority – junior  – senior | 4.25  3.96 | 1.87  1.94 | 4.61  4.61 | 1.78  1.85 | 4.41  4.25 | 1.62  1.93 |
| 2 | Stance – accommodative  – nonaccommodative | 3.70  5.27 | 1.92  1.48 | 3.14  5.08 | 1.64  1.46 | 4.87  3.76 | 1.38  1.51 |
|  | Discipline – nurse/midwife  – allied health  – medical officers | 4.72  4.50  4.24 | 1.86  1.79  1.97 | 4.18  4.24  3.90 | 1.72  1.91  1.81 | 4.39  3.84  4.71 | 1.62  1.58  1.63 |
|  | Seniority – junior  – senior | 4.40  4.57 | 1.90  1.86 | 4.18  4.03 | 1.66  1.94 | 4.29  4.35 | 1.63  1.62 |
| 3 | Stance – accommodative  – nonaccommodative | 3.08  3.12 | 1.72  1.87 | 3.28  3.14 | 1.82  1.71 | 2.86  2.92 | 1.72  1.38 |
|  | Discipline – nurse/midwife  – allied health  – medical officers | 3.08  2.91  3.31 | 1.70  1.76  1.92 | 3.13  3.50  2.99 | 1.89  1.73  1.68 | 3.24  2.50  2.98 | 1.60  1.34  1.63 |
|  | Seniority – junior  – senior | 2.87  3.33 | 1.69  1.87 | 3.19  3.22 | 1.76  1.78 | 2.76  3.01 | 1.54  1.56 |
| 4 | Stance – accommodative  – nonaccommodative | 3.34  3.43 | 1.92  1.98 | 3.51  3.76 | 2.10  1.87 | 2.90  3.68 | 1.77  1.65 |
|  | Discipline – nurse/midwife  – allied health  – medical officers | 3.34  3.30  3.50 | 1.76  1.93  2.16 | 3.70  3.83  3.37 | 2.00  1.96  2.00 | 3.71  3.10  3.06 | 1.93  1.68  1.60 |
|  | Seniority – junior  – senior | 3.21  3.56 | 1.84  2.04 | 3.52  3.75 | 1.99  1.97 | 3.12  3.46 | 1.84  1.63 |
| 5 | Stance – accommodative  – nonaccommodative | 5.36  5.38 | 1.51  1.51 | 5.53  5.63 | 1.42  1.35 | 5.28  5.46 | 1.71  1.44 |
|  | Discipline – nurse/midwife  – allied health  – medical officers | 5.51  5.23  5.38 | 1.38  1.69  1.43 | 5.34  5.72  5.69 | 1.52  1.35  1.27 | 5.84  5.21  5.06 | 1.67  1.33  2.06 |
|  | Seniority – junior  – senior | 5.28  5.47 | 1.51  1.50 | 5.40  5.76 | 1.59  1.10 | 5.45  5.29 | 1.43  1.71 |
| 6 | Stance – accommodative  – nonaccommodative | 5.88  5.89 | 1.30  1.10 | 6.36  5.99 | 0.99  1.23 | 5.96  5.24 | 1.30  1.64 |
|  | Discipline – nurse/midwife  – allied health  – medical officers | 5.88  5.80  5.97 | 1.25  1.21  1.14 | 6.04  6.09  6.39 | 1.17  1.19  1.01 | 5.67  5.60  5.52 | 1.50  1.64  1.51 |
|  | Seniority – junior  – senior | 6.05  5.72 | 1.04  1.33 | 6.17  6.18 | 1.16  1.10 | 5.32  5.87 | 1.67  1.36 |
| 7 | Stance – accommodative  – nonaccommodative | 3.89  3.78 | 1.79  1.83 | 3.58  3.99 | 2.07  1.96 | 3.73  4.06 | 1.75  1.75 |
|  | Discipline – nurse/midwife  – allied health  – medical officers | 3.69  3.90  3.93 | 1.77  1.87  1.78 | 3.95  4.06  3.34 | 2.01  2.04  1.99 | 4.05  3.79  3.83 | 1.73  1.93  1.67 |
|  | Seniority – junior  – senior | 3.84  3.84 | 1.82  1.80 | 3.95  3.62 | 2.08  1.96 | 3.70  4.08 | 1.81  1.69 |
| 8 | Stance – accommodative  – nonaccommodative | 2.89  3.05 | 1.70  1.69 | 2.33  2.84 | 1.40  1.89 | 1.95  2.10 | 1.07  1.41 |
|  | Discipline – nurse/midwife  – allied health  – medical officers | 2.89  3.20  2.81 | 1.62  1.86  1.58 | 2.45  2.70  2.60 | 1.62  1.85  1.60 | 1.88  1.96  2.23 | 1.35  1.23  1.23 |
|  | Seniority – junior  – senior | 2.95  2.99 | 1.69  1.70 | 2.45  2.71 | 1.47  1.87 | 1.98  2.07 | 1.28  1.24 |
| 9 | Stance – accommodative  – nonaccommodative | 4.47  4.76 | 1.92  1.83 | 5.39  5.02 | 1.83  1.87 | 4.24  4.68 | 2.12  2.02 |
|  | Discipline – nurse/midwife  – allied health  – medical officers | 4.63  4.80  4.42 | 1.87  1.83  1.95 | 5.47  5.19  4.96 | 1.82  1.80  1.95 | 4.46  4.60  4.31 | 2.06  2.13  2.11 |
|  | Seniority – junior  – senior | 4.44  4.79 | 1.91  1.84 | 5.34  5.07 | 1.71  2.01 | 4.44  4.47 | 2.02  2.14 |
| 10 | Stance – accommodative  – nonaccommodative | 4.68  5.00 | 1.55  1.40 | 5.09  4.73 | 1.58  1.60 | 5.15  5.08 | 1.36  1.40 |
|  | Discipline – nurse/midwife  – allied health  – medical officers | 4.79  4.95  4.77 | 1.45  1.48  1.52 | 4.89  4.88  4.96 | 1.60  1.41  1.77 | 4.87  5.35  5.12 | 1.48  1.08  1.51 |
|  | Seniority – junior  – senior | 4.93  4.75 | 1.46  1.50 | 4.98  4.84 | 1.45  1.72 | 5.08  5.15 | 1.36  1.40 |
| *M: Mean; SD: Standard Deviation* | | | | | | | |
